# Effect of Parental Myopia on Change in Refraction in Shanghai Preschoolers: A 1-Year Prospective Study

**DOI:** 10.3389/fped.2022.864233

**Published:** 2022-04-25

**Authors:** Yingyan Ma, Senlin Lin, Jianfeng Zhu, Rong Zhao, Bo Zhang, Yao Yin, Yueqin Shao, Xiangui He, Xun Xu, Haidong Zou

**Affiliations:** ^1^Department of Preventative Ophthalmology, Shanghai Eye Disease Prevention and Treatment Center, Shanghai Eye Hospital, Shanghai, China; ^2^Department of Ophthalmology, Shanghai General Hospital, School of Medicine, Shanghai Jiao Tong University, Shanghai, China; ^3^Shanghai Shenkang Hospital Development Center, Shanghai, China; ^4^Jiading Center for Disease Prevention and Control, Shanghai, China

**Keywords:** preschool children, refraction, axial length, parental myopia, outdoor activities

## Abstract

**Background:**

To investigate the risk factors for change in refraction and refractive components in preschoolers.

**Methods:**

Preschool children aged 3–5 years old, from the junior and the middle grades of seven randomly selected kindergartens in Jia Ding District, Shanghai, were followed for 1 year. Cycloplegic autorefraction (1% cyclopentolate) and axial length (AL) were measured at baseline and at 1-year follow-up. Questionnaires about parental myopia and environmental risk factors, such as time of outdoors and near work, were collected.

**Results:**

A total of 603 right eyes of 603 children were included. Parental myopia was not associated with a change in refraction, but two myopic parents were associated with a longer change in AL (coefficient = 0.153, *p* = 0.006), after adjusted for baseline spherical refraction, age, gender, change in height, change in weight, and environment risk factors. In the multivariate analyses, boys showed a more myopic refraction shift than girls in 1 year (coefficient = −0.150, *p* = 0.008) and a quicker AL elongation (coefficient = 0.120, *p* = 0.008). Time of near work, such as watching television, using computer, reading and writing, and time of outdoor activities, was not associated with a change in refraction or AL.

**Conclusions:**

In preschool age, environmental risk factors were not strongly associated with the change in refraction or refractive components. Parental myopia influences the refractive development of children continuously from infancy to preschool age, which might be the biological basis of school myopia.

## Introduction

Risk factors for myopia have been comprehensively studied, such as parental myopia, time spent in near work and outdoor activities, and intensive education (1–3). Generally, the prevalence and incidence of myopia in preschool children were quite low, despite a progressively increasing prevalence of myopia reported in high myopia prevalence regions, such as Singapore and Taiwan (4, 5). The presence of myopia at the entrance of the primary school was an important risk factor for the incidence of high myopia in the teenage and adulthood (6, 7). In addition, the refraction [spherical equivalent (SE)] at school entrance also largely determined the incidence of school myopia (8, 9). Therefore, exploring risk factors for change in refraction and refractive components in preschool children was valuable.

Studies about risk factors for refractive change or refractive errors for preschool children were limited, among which, most of the studies were cross-sectional (10–14). The reported risk factors for myopia in preschoolers included parental myopia, ethnic group, younger age (<36 months), smoking mother at pregnant or passive smoking 6 months before birth, and astigmatism (10–13, 15). However, childhood exposure to passive smoke was not associated with the mean values for SE or axial length (AL) change (15). Time spent on near work or on outdoor activities was not found to be the risk factor for myopia prevalence in 3-year-old children (10, 11). However, one study reported a close relationship between outdoor time at 3 years old and future myopia in teenage years (16). Birth season was also reported to be related to refraction in very young children, however, the relationship is controversial and might be very subtle (17, 18).

For children aged 3–6 years old, the change in SE and the prevalence of the different types of refractive errors were quite stable at this stage (19, 20). In addition, it is reported that for myopic children, refraction stability and even regression were observed in a significant proportion of preschool children (21, 22), which indicated the special characteristics of the refractive development in the preschoolers. Therefore, longitudinal studies were needed to explore the associated risk factors for the change in refraction and refractive components in the preschoolers.

## Methods

### Study Design and Participants

Taking kindergarten as a unit, random cluster sampling was conducted on all kindergartens in Jia Ding District, Shanghai, by lottery method. Seven kindergartens were randomly selected, and all the preschool children aged 3–5 years old from the junior and the middle grades in the selected kindergartens were included in the study. Children were followed for 1 year. The first visit was carried out from November to December in 2014; the second visit was carried out from November to December in 2015. Considering the length of the schooling system of kindergartens in Shanghai, children of senior grade level were not included, because those children will enter primary schools after 1 year and will be lost to follow-up.

The research protocol was adhered to the Declaration of Helsinki for research that involves human subjects. Written informed consent was signed by either a parent or legal guardian of each subject, and oral agreement was obtained from the children themselves. Children and parents could choose whether to accept cycloplegia or not. Only those who agreed to cycloplegia received 1% cyclopentolate to dilate pupils. The study was approved by the Ethics Committee (No. 2015KY150). Children with written informed consent were included in the study. Those with severe vision-threatening diseases, such as congenital cataract or severe ocular trauma, were excluded from the study.

### Measures

For cycloplegia, children were first anesthetized topically with one drop of 0.5% proparacaine hydrochloride in each eye. After about 15 s, cycloplegia was induced with two drops of 1% cyclopentolate (Cyclogyl; Alcon, Fort Worth, TX, USA) 5 min apart in each eye. Pupil size and light reflex were examined 30 min after the first drops of cyclopentolate, and if the pupil was dilated to 6 mm or larger and a light reflex was absent, cycloplegia was deemed completely. If the pupillary light reflex was still present, the third drop of cyclopentolate was given 15 s after another drop of proparacaine hydrochloride in each eye. Children, who still failed the standards for successful cycloplegia 15–20 min later, were not given more eye drops, and were recorded as unsuccessful cycloplegia.

Axial length was measured with three consecutive measurements (IOL Master, version 5.02, Carl Zeiss, Jena, Germany) and the average reading of the three measurements was calculated. If the differences in any of the two measurements were larger than 0.02 mm, a further measurement was conducted. Similarly, the average of three consecutive auto-refractor (KR-8900, Topcon, Tokyo, Japan) measurements after successful cycloplegia was used for determining the spherical diopters (DSs), cylinder diopters (DCs), and corneal curvature radius (CR) of the children. If any of the two measurements varied by more than 0.50 diopters (D), a further measurement was taken. Every day before the examination, the machines were all preheated for several minutes, and the auto-refractor and the IOL Master were calibrated using a model eye.

### Questionnaires

Risk factors were collected through questionnaires. The questionnaires were filled by parents or guardians at baseline and follow-up. Parental myopia, average eye usage time at home and school on weekdays and weekends during the most recent month were collected. Time spent on reading and writing, using tablet computer and mobile phone, playing computer, watching television, and time spent outdoors was investigated.

### Statistical Analyses

Since the Pearson coefficients were 0.90 and 0.94 for the baseline SE and AL between the right and the left eyes (both *p* < 0.001) and 0.90 and 0.96 for the follow-up SE and AL (both *p* < 0.001), the right eye was used for data analyses. One year change in refraction and refractive components was calculated as follows: measurement at the second visit—measurement at baseline. The SE equals to DS + 0.5 × DC. The eye usage time per day was calculated as the (time at weekdays × 5 + time at weekend × 2)/7. Eye usage time was classified into three categories according to the tertiles. Since about 63.2% of children spent ≤ 0.25 h per day on the computer, the time spent on the computer was classified into two categories as low (≤0.25 h) and high (>0.25 h). The time spent in reading, writing, and using mobile phone and tablet computer was merged together as the time spent on reading and writing, since the working distances were similar among those near work activities.

Univariate and multivariate linear regression analyses were performed to investigate the associations between risk factors and baseline SE, baseline AL, change in SE, and change in AL. The change in SE was further classified into ≥0.25 D, <0.25 and ≥0.1 D, <0.1 and ≥-0.1 D, <-0.1 and ≥-0.25 D, and <-0.25 D, and proportions of change in SE were compared between children with different number of myopic parents using a chi-square test. SAS 9.4 (SAS Institute, Cary, NC, USA) and SPSS 22.0 (IBM SPSS Inc. Chicago, IL, USA) were used for statistical analyses. A value of *p* < 0.05 was considered statistically significant.

## Results

At the first visit, a total of 903 children were successfully cyclopleged. Among them, a total of 857 (94.9%) children were re-examined 1 year later, at which time 613 children (67.9%) were successfully cyclopleged, and 603 children with right eye data completed. Therefore, a total of 603 right eyes of 603 children were included in the analyses. The included and excluded children did not differ significantly with respect to baseline age (*p* = 0.223), gender (*p* = 0.763) or SER (spherical equivalent refraction) (*p* = 0.348), and AL (*p* = 0.246) of their right eyes. The baseline characteristics of the study population are listed in Table 1.

**Table 1:** Basic characteristics of the study population as a function of age.

		**3 years old**	**4 years old**	**5 years old**	**All**	*P-*value^*^
No.		159	332	112	603	
Gender (girls, %)		40.9	45.8	53.6	45.9	0.118
LogMAR VA at 1st visit (Mean ± SD)		0.24 ± 0.10	0.21 ± 0.10	0.17 ± 0.09	0.21 ± 0.10	<0.001
LogMAR VA at 2nd visit (Mean ± SD)		0.20 ± 0.11	0.16 ± 0.09	0.15 ± 0.12	0.17 ± 0.11	<0.001
Baseline myopia prevalence (%)		1.9	1.5	0	1.3	0.374
SE change (*D*, mean ± SD)		0.008 ± 0.50	−0.004 ± 0.47	−0.008 ± 0.46	−0.001 ± 0.47	0.988
AL change (mm, mean ± SD)		0.32 ± 0.21	0.22 ± 0.34	0.23 ± 0.46	0.25 ± 0.34	0.014
Change in height (cm, mean ± SD)		7.09 ± 1.15	7.29 ± 2.40	7.02 ± 2.08	7.18 ± 2.07	0.528
Change in weight (kg, mean ± SD)		2.46 ± 1.15	2.80 ± 1.58	2.96 ± 1.68	2.74 ± 1.50	0.051
Number of myopic parents (%)	0	35.14	36.11	41.82	36.94	0.620
	1	41.22	38.27	31.82	37.80	
	2	23.65	25.62	26.36	25.26	
TV (h, mean ± SD)		1.33 ± 0.88	1.38 ± 0.77	1.42 ± 0.84	1.37 ± 0.81	0.667
Read and write (h, mean ± SD)		1.59 ± 0.74	1.74 ± 0.99	1.78 ± 0.84	1.71 ± 0.91	0.184
Outdoors (h, mean ± SD)		2.82 ± 1.74	2.84 ± 1.40	2.86 ± 1.59	2.84 ± 1.53	0.985
Computer (h, mean ± SD)		0.47 ± 0.49	0.44 ± 0.36	0.45 ± 0.37	0.45 ± 0.40	0.826

*SD, standard deviation; D, diopter*.

* *One-way ANOVA was used to compare differences of continuous variables between various age groups. The chi-square test was used to compare differences of categorical variables between various age groups*.

At baseline, the prevalence of myopia (SE ≤ −0.5 D), astigmatism (DC ≤ −2.0 D), and hyperopia (≥+3.0 D) were 1.33% (8/603), 2.32% (14/603), and 2.82% (17/603). The 1-year incidence rates of myopia were 1.92% (3/156), 0.92% (3/327), and 0.89% (1/112) in the 3-, 4-, and 5-year olds. The 1-year incidence rates of astigmatism were 3.18% (5/157), 0.93% (3/324), and 0% (0/108) in the 3-, 4-, and 5-year olds. The 1-year incidence rates of hyperopia were 1.96% (3/153), 0.61% (2/326), and 0.93% (1/107) in the 3-, 4-, and 5-year olds.

The average changes of SE were 0.34 D [standard deviation (SD) = 0.58], −0.003 D (SD = 0.47), and −0.13 D (SD = 0.58) for those who were suffering from myopia, emmetropia (−0.5 D < SE <3 D), and hyperopia at baseline, and the median values were 0.375, 0, and −0.13 D, respectively. The average changes of AL were 0.31 mm (SD = 0.10), 0.25 mm (SD = 0.35), and 0.32 mm (SD = 0.31) for those who were suffering from myopia, emmetropia, and hyperopia at baseline, and the median values were 0.27, 0.26, and 0.23 mm, respectively.

**Figure 1:**
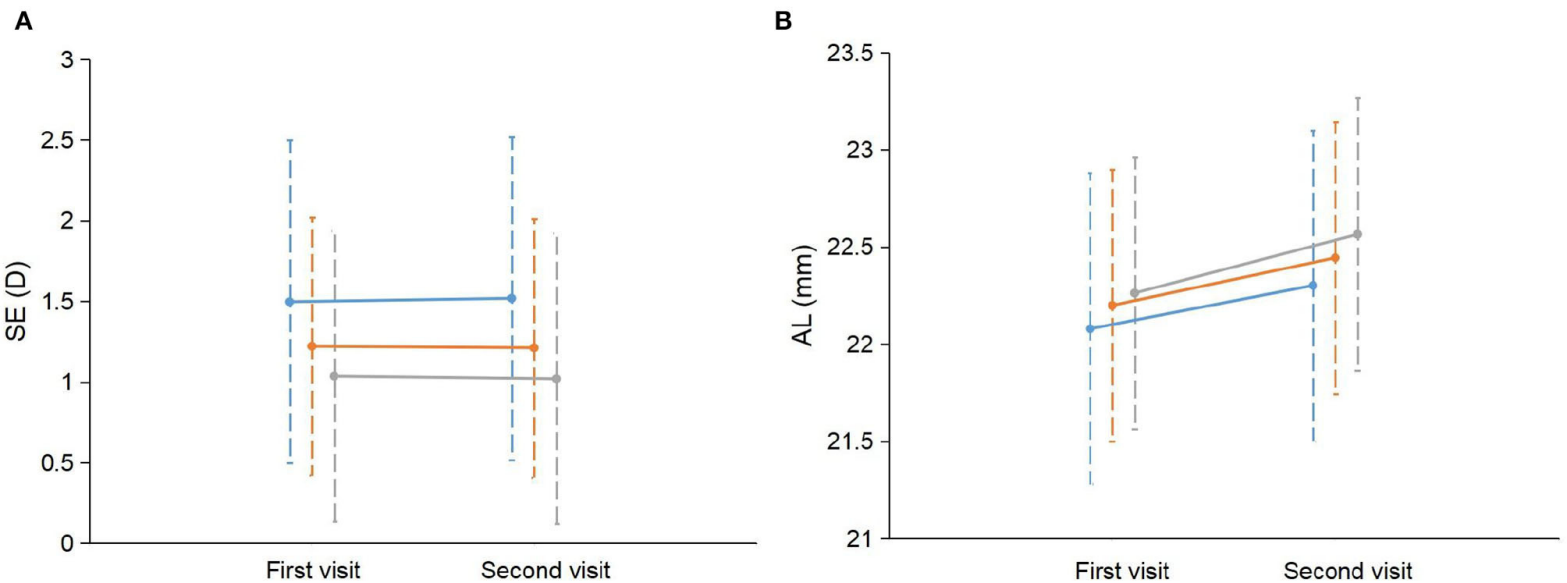
Change in SE and AL between children with different number of myopic parents. **(A)** The mean SE and standard deviation (SD) at the first visit and the second visit were plotted separately for children with 0, 1, and 2 myopic parents. **(B)** The mean AL and SD at the first visit and the second visit were plotted separately for children with 0, 1, and 2 myopic parents. The blue line represents children with no myopic parents. The orange line represents children with one myopic parent. The gray line represents children with two myopic parents. SE, spherical equivalent refraction; AL, axial length.

**Figure 2:**
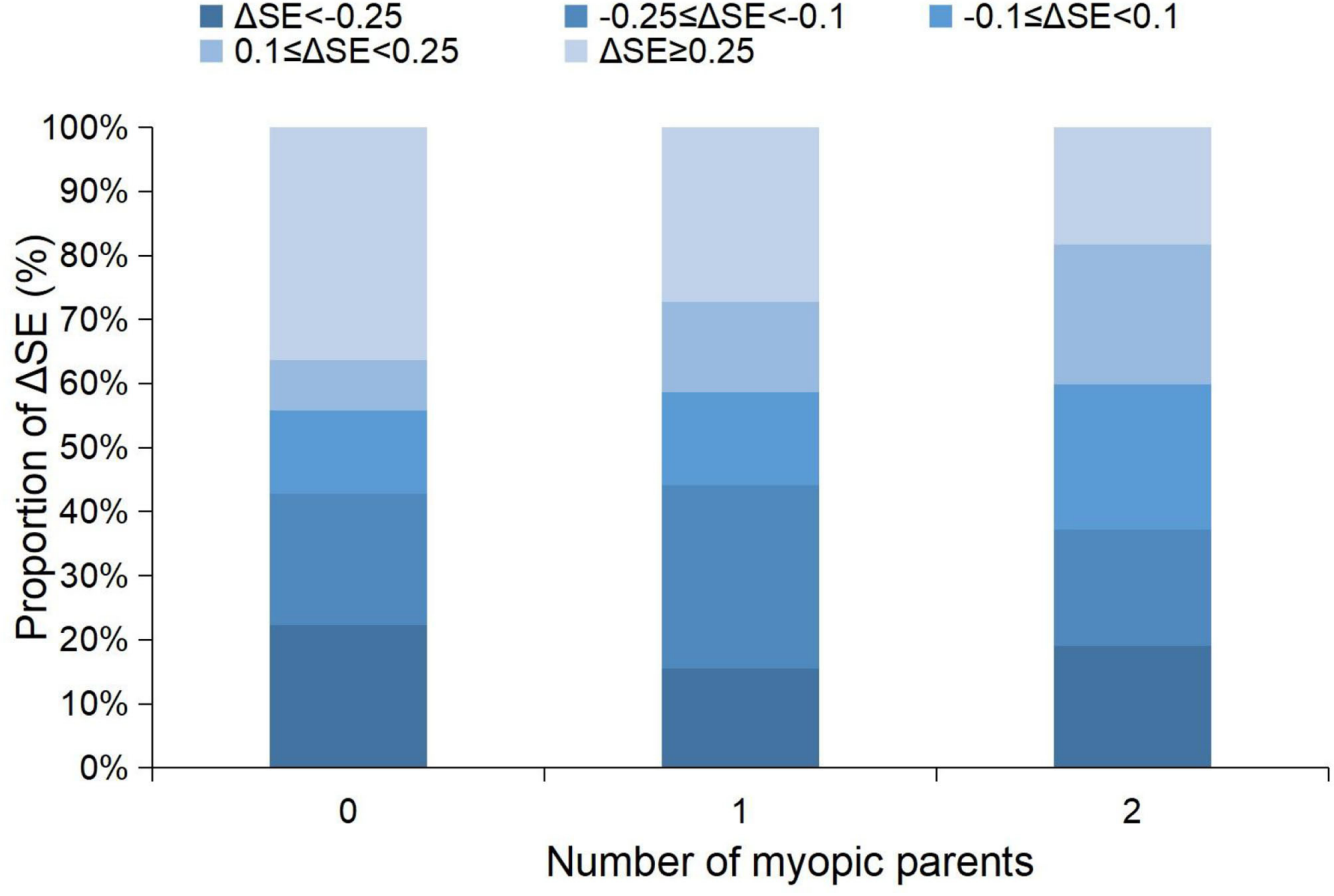
Stacked histogram of the 1-year changes in SE grouped by the amplitude of the changes between children with different numbers of myopic parents. SE, spherical equivalent refraction.

For the change in SE, the univariate analyses showed that boys (coefficient = −0.102, *p* = 0.009) and a more hyperopic baseline SE (coefficient = −0.076, *p* < 0.001) were associated with a more myopic shift in SE; larger change in height (coefficient = 0.024, *p* = 0.03) was associated with a more hyperopic shift in SE (Table 2). The multivariate analyses showed that boys and a more hyperopic baseline SE were associated with a change in SE toward the myopic direction (Table 3). Parental myopia and the environmental risk factors were not significantly associated with the change in SE (Tables 2, 3; Figure 1). Although the proportion of hyperopic shift of SE ≥ 0.25 D was decreased with the increased number of myopic parents (Figure 2), the proportions of change in SE were not statistically different between children with 0, 1, or 2 myopic parents (*X*^2^ = 13.94, *p* = 0.08).

**Table 2:** Univariate regression analyses for the change in SE and the change in AL^*^.

		**Change in SE**		**Change in AL**	
		**β**	***P-*value**	**β**	***P-*value**
Age		−0.004 (−0.061, 0.053)	0.89	−0.050 (−0.092, −0.009)	0.02
Gender	F	Ref		Ref	
	M	−0.102 (−0.178, −0.026)	0.009	0.055 (0.000, 0.110)	0.05
Baseline SE		−0.076 (−0.116, −0.035)	<0.001	0.015 (−0.015, 0.044)	0.33
Change in height		0.024 (0.002, 0.046)	0.03	0.015 (−0.002, 0.032)	0.08
Change in weight		0.013 (−0.018, 0.043)	0.41	−0.003 (−0.026, 0.021)	0.82
Parental myopia	0	Ref		Ref	
	1	−0.031 (−0.121, 0.059)	0.50	0.018 (−0.047, 0.083)	0.58
	2	−0.038 (−0.138, 0.062)	0.46	0.085 (0.013, 0.158)	0.02
Computer	≤0.25	Ref		Ref	
	>0.25	−0.038 (−0.130, 0.055)	0.42	−0.026 (−0.096, 0.045)	0.47
TV (hrs)	≤0.97	Ref		Ref	
	≤1.43	−0.011 (−0.112, 0.091)	0.84	−0.006 (−0.082, 0.071)	0.89
	>1.43	−0.044 (−0.141, 0.053)	0.38	−0.048 (−0.121, 0.025)	0.20
Read and write (hrs)	≤1.18	Ref		Ref	
	≤1.82	−0.036 (−0.137, 0.066)	0.49	0.053 (−0.023, 0.129)	0.17
	>1.82	−0.037 (−0.138, 0.064)	0.47	0.010 (−0.066, 0.086)	0.79
Outdoors (hrs)	≤2.1	Ref		Ref	
	≤3.1	−0.013 (−0.110, 0.085)	0.80	0.025 (−0.047, 0.097)	0.49
	>3.1	0.053 (−0.045, 0.151)	0.29	−0.039 (−0.112, 0.033)	0.29

*SE, spherical equivalent refraction; AL, axial length*.

* *Univariate linear regression analyses were used, and beta coefficients and the 95% confidence interval (CI) were presented in the table*.

**Table 3:** Multivariate linear regression models for change in SE^*^.

**Variables**		**Model 1**		**Model 2**		**Model 3**	
		**β**	***P-*value**	**β**	***P-*value**	**β**	***P-*value**
Age		0.035 (−0.046, 0.117)	0.393	0.035 (−0.048, 0.117)	0.406	0.041 (−0.042, 0.124)	0.331
Gender	F	Ref	/	Ref	/	Ref	/
	M	−0.157 (−0.226, −0.047)	0.005	−0.154 (−0.264, −0.044)	0.006	−0.150 (−0.261, −0.040)	0.008
Baseline SE		−0.148 (−0.212, −0.085)	<0.001	−0.150 (−0.213, −0.087)	<0.001	−0.153 (−0.217, −0.090)	<0.001
Parental myopia	0	Ref		Ref	/	Ref	/
	1	−0.081 (−0.208, 0.046)	0.212	−0.066 (−0.194, 0.062)	0.312	−0.064 (−0.192, 0.064)	0.329
	2	−0.129 (−0.266, 0.007)	0.063	−0.115 (−0.252, 0.022)	0.099	−0.118 (−0.255, 0.020)	0.093
Change in height				0.024 (−0.006, 0.054)	0.110	0.025 (−0.005, 0.055)	0.101
Change in weight				0.002 (−0.042, 0.046)	0.929	0.007 (−0.038, 0.051)	0.771
Computer	0					Ref	/
	1					−0.082 (−0.197, 0.033)	0.160
TV	0					Ref	/
	1					−0.005 (−0.143, 0.134)	0.946
	2					−0.058 (−0.195, 0.079)	0.409
Reading	0					Ref	/
	1					−0.123 (−0.256, 0.010)	0.069
	2					−0.118 (−0.261, 0.025)	0.106
Outdoor	0					Ref	/
	1					0.036 (−0.098, 0.170)	0.597
	2					0.083(−0.058, 0.225)	0.247

*SE, spherical equivalent refraction*.

* *Multivariate linear regression analyses were used, and beta coefficients and the 95% confidence interval (CI) were presented in the table. Model 1 is adjusted for age, gender, baseline SE, and parental myopia, and the value of p is <0.001 for the model. Model 2 is adjusted for age, gender, baseline SE, parental myopia, and change in height and weight, and the value of p is <0.001 for the model. Model 3 is adjusted for age, gender, baseline SE, parental myopia, change in height and weight, and environmental risk factors, and the value of p is 0.001 for the model*.

For the change in AL, the univariate analysis showed that two myopic parents (coefficient = 0.085, *p* = 0.02) and younger age (coefficient = −0.050, *p* = 0.02) were associated with more rapid AL elongation (Table 2; Figure 1). The multivariate analysis showed that more hyperopic baseline SE, two myopic parents, younger age, boys, and greater change in height were associated with more rapid elongation in AL (Table 4). The environmental risk factors were not significantly associated with the change in AL (Tables 2, 4).

**Table 4:** Multivariate linear regression models for change in AL^*^.

**Variables**		**Model 1**		**Model 2**		**Model 3**	
		**β**	***P-*value**	**β**	***P-*value**	**β**	***P-*value**
Age		−0.078 (−0.145, −0.012)	0.020	−0.073 (−0.139, −0.007)	0.029	−0.067 (−0.133, −0.001)	0.047
Gender	F	Ref		Ref		Ref	
	M	0.096 (0.007, 0.185)	0.034	0.106 (0.018, 0.194)	0.018	0.120 (0.032, 0.208)	0.008
Baseline SE		0.074 (0.023, 0.125)	0.005	0.073 (0.023, 0.123)	0.005	0.079 (0.029, 0.130)	0.002
Parental myopia	0	Ref		Ref		Ref	
	1	0.070 (−0.033, 0.172)	0.183	0.092 (−0.009, 0.194)	0.075	0.090 (−0.011, 0.191)	0.082
	2	0.126 (0.016, 0.236)	0.026	0.146 (0.037, 0.255)	0.009	0.153 (0.044, 0.262)	0.006
Change in height				0.041(0.018, 0.065)	0.001	0.041 (0.018, 0.065)	0.001
Change in weight				−0.018 (−0.053, 0.017)	0.320	−0.015 (−0.050, 0.021)	0.420
Computer	0					Ref	
	1					−0.063 (−0.154, 0.028)	0.174
TV	0					Ref	
	1					0.035 (−0.074, 0.145)	0.527
	2					−0.089 (−0.197, 0.019)	0.107
Reading	0					Ref	
	1					0.041 (−0.064, 0.146)	0.445
	2					0.064 (−0.049, 0.177)	0.266
Outdoor	0					Ref	
	1					0.071 (−0.035, 0.177)	0.189
	2					0.041 (−0.071, 0.153)	0.471

*AL, axial length; SE, spherical equivalent refraction*.

* *Multivariate linear regression analyses were used, and beta coefficients and the 95% confidence interval (CI) were presented in the table. Model 1 is adjusted for age, gender, baseline SE, and parental myopia, and the value of p is 0.001 for the model. Model 2 is adjusted for age, gender, baseline SE, parental myopia, and change in height and weight, and the value of p is <0.001 for the model. Model 3 is adjusted for age, gender, baseline SE, parental myopia, change in height and weight, and environmental risk factors, and the value of p is <0.001 for the model*.

## Discussion

In the present study, environmental risk factors, such as the time of near work and outdoor activities, were not associated with the change in SE or AL in the 1-year follow-up period. Parental myopia was not associated with the change in SE, but associated with the change in AL. The change in SE did not show the difference with the increasing age, however, the elongation in AL was decreased with age. Boys showed a quicker AL elongation than girls and were more likely to have a myopic SE change.

### Environmental Risk Factors and Refractive Development

Animal studies suggested that the visual environment plays an important role in the ocular growth and refractive development (23). In animal models, form deprivation could cause myopia, and lens- induced myopic defocus/hyperopic defocus could lead to hyperopia or myopia (24, 25). In young humans, visual deprivation from congenital cataract, ptosis, corneal opacity, or other ocular diseases could lead to the failure of emmetropization and development of myopia (26, 27). For school-aged children, time outdoors were regarded as protective factors for incident myopia, probably because of the intensive light level in outdoor settings (28).

Studies in preschool children were limited, and most were cross-sectional and did not observe strong related factors (10–13, 15–18). In the present study, time of near work and outdoor activities were not associated with refraction or refractive change, probably because the variation of the activity time was small among the young children. Another explanation is that the refraction of children during this age period was not sensitive to the environmental risk factors and therefore, remains quite stable with very low incidence or prevalence of refractive errors.

In the present cohort, most children spent <2 h on the reading (such as using mobile phone and tablet computer) and writing per day, about 1 h on watching television, and <0.25 h on using the computer. Most children spent around 2 h per day on outdoor activities, and some even reached 3 h a day. These could be the most important reasons why the incidence of myopia was relatively low in the preschoolers. On the contrary, the school-aged children in Shanghai were reported to spend about 1 h a day on outdoor activities, and much more time in near work-related activities apart from having lessons at school (9). The change in the environment associated with much longer eye usage time and limited outdoor time could be the major reason for epidemics of myopia in school-aged children (29).

### Parental Myopia and Refractive Development

Previous studies suggested that early-onset myopia was closely related to genetics (10, 11, 30, 31). Using umbilical cord tissues, *in-utero* epigenetic factors were found to be related to myopia onset before 3 years old (31). Cross-sectional studies indicated that children with two myopic parents were more likely to be myopic at the age between 6 and 72 months and were more likely to have a less hyperopic SE and longer AL when compared with children without myopic parents (10, 11, 30). Children with tall height were also associated with a more myopic SE and longer AL in children younger than 6 years old (10, 11). Tideman et al. showed that weight and height during pregnancy and 2 years postnatally were closely associated with AL and corneal CR at 4.9–9 years old, which indicated the genetic basis for the development of refractive components (32).

An interesting finding was that parental myopia was not significantly associated with the 1-year changes in SE in the present children aged 3–5 years old. Further analyses showed that the changes in SE were 0.022 D (SD = 0.514), −0.014 D (0.470), and −0.030 D (0.420) for two myopic parents, one myopic parent, and none myopic parent, respectively. Although a myopic trend was observed in terms of the mean value, the difference was not statistically significant or was clinically relevant. On the contrary, the multivariate analysis showed that children with two myopic parents when compared with no myopic parents had a quicker elongation of AL by 0.15 mm, which was similar to those reported in the Population-Based Pediatric Eye Disease Study (POPEYE) (30). The difference also had clinical meaning, which was equivalent to about 0.3 D in refraction. Parental myopia was closely related to the baseline SE (not presented in the results), those with two myopic parents had a mean SE of 0.37 D [95% confidence interval (CI): 0.18–0.57, *p* < 0.001] more myopic than those without myopic parents, which was similar to what was reported in Singapore and the POPEYE (about 0.35–0.39 D SE difference between two myopic parents and no myopic parents) (11, 30).

The reason why the growth of the AL did not show a difference in SE could be that it was mainly compensated by changes in other refractive components, such as a decrease in crystalline lens power. But once these compensatory factors were exhausted, children with two myopic parents were more susceptible to suffer from myopia when compared with children without myopic parents. The results indicated a continuous effect of parental myopia on AL and SE, not only in the early period after birth, but also in preschool children aged 3–6 years old. This may be the biological basis of the influence of parental myopia on the incidence of myopia in school-age children.

### Gender and Refractive Development

In preschool children aged 3–6 years old, boys showed more rapid elongation of AL than girls (nearly 0.1 mm quicker than girls) and a more myopic change in SE (about 0.135 D more myopic than girls). However, at school-aged children, girls were usually at higher risk of myopia than boys. A meta-analysis revealed that after 9 years old age, gender difference begins to emerge in White and East Asians, and the difference becomes more obvious with increasing age, with girls being twice as likely as boys to be myopic at the age of 18 (33).

The phenomenon could be partially explained by the difference in the growth rate of height between the two genders. According to the growth curves of Chinese children, before 10 years old, the median height of boys was longer than that of girls, however, after 10 years old, the median height of girls exceeds that of boys. Then after 12 years old, the boys again showed longer height than girls (34).

However, even adjusted for change in height, boys still showed quicker elongation of AL than girls in the preschoolers (Table 4, Models 2 and 3), suggesting that there are other mechanisms in gender regulation of refraction and AL growth.

### Strength and Limitation

There were several limitations in the present study. First, the sample size is not large enough to include the adequate number of incident myopia, therefore, the risk factors for incident myopia cannot be determined in the present study. Second, the percentage of the loss of follow-up was relatively high. Although comparisons of baseline characteristics were not significantly different between those who were followed and those who were lost, cautions should be paid when interpreting the results, considering possible bias. Third, the use of cycloplegic autorefraction without retinoscopy could cause bias in the estimation of the true value of refraction for preschoolers. Studies have found that although the mean difference between cycloplegic autorefraction and retinoscopy could be <0.25 D, the variance could be as large as 3.5 D in either refractive direction (35). The use of a table-mounted auto-refractor in the present study was reported to be more accurate when compared with a hand-held auto-refractor, reducing the errors to some extent (36). Last but not the least, the environmental risk factors were collected through questionnaires, which could be imprecise due to recall bias. A recent study pointed out that using questionnaires could probably overestimate the time of near work (37). Future research can use more accurate and objective measurement methods, such as light measuring wearable devices, to determine the exposure of children to environmental risk factors, so as to obtain more accurate results.

The strength of the present study included the longitudinal design and the use of cycloplegia to obtain accurate refraction in the preschoolers. The present study revealed the change in refraction and refractive components with age and gender in the age between 3 and 5 years old. The study results also explained the reason that parental myopia could be an important risk factor for incident myopia in school-aged children. Exposure to myopia-related environmental risk factors was limited in the present population, therefore, protecting the preschoolers from being myopic.

## Data Availability Statement

The raw data supporting the conclusions of this article will be made available by the authors, without undue reservation.

## Ethics Statement

The studies involving human participants were reviewed and approved by Ethics Committee of Shanghai General Hospital. Written informed consent to participate in this study was provided by the participants' legal guardian/next of kin.

## Author Contributions

YM and SL analyzed the data, wrote the manuscript, and participated in the design of the study. JZ and RZ collected data and helped to interpret the data. BZ, YY, and YS helped to analyze the data and interpret the data. XX, XH, and HZ designed the study and revised the manuscript. All authors contributed to the article and approved the submitted version.

## Funding

Financial support for this study was provided by the Chinese National key research and development program (Project number 2021YFC2702100), The Science and Technology Commission of Shanghai Municipality (Project No. 20DZ1100200), Shanghai Municipal Commission of health (public health system three-year plan-Key Subjects) (Project No. GWV10.1-XK06), the Project of Shanghai Shen Kang Hospital Development Centre (Grant No. SHDC2020CR30538, SHDC2018110, SHDC12021613) Shanghai engineering research center of precise diagnosis and treatment of eye diseases, Shanghai, China (Project No. 19DZ2250100), Chinese Natural Science Foundation for Young Staff (No. 81800881), Shanghai Sailing Program (No. 17YF1416100), and Shanghai Municipal Health Commission (No. 20184Y0217). The funding agreement ensured the authors' independence in designing the study, interpreting the data, writing, and publishing the report.

## Conflict of Interest

The authors declare that the research was conducted in the absence of any commercial or financial relationships that could be construed as a potential conflict of interest.

## Publisher's Note

All claims expressed in this article are solely those of the authors and do not necessarily represent those of their affiliated organizations, or those of the publisher, the editors and the reviewers. Any product that may be evaluated in this article, or claim that may be made by its manufacturer, is not guaranteed or endorsed by the publisher.
